# Multisystem mitochondrial diseases due to mutations in mtDNA-encoded subunits of complex I

**DOI:** 10.1186/s12887-020-1912-x

**Published:** 2020-01-29

**Authors:** Tereza Danhelovska, Hana Kolarova, Jiri Zeman, Hana Hansikova, Manuela Vaneckova, Lukas Lambert, Vendula Kucerova-Vidrova, Kamila Berankova, Tomas Honzik, Marketa Tesarova

**Affiliations:** 1 Department of Pediatrics and Adolescent Medicine, First Faculty of Medicine, Charles University and General University Hospital in Prague, Ke Karlovu 2, 128 08 Praha 2, Prague, Czech Republic; 2 Department of Radiology, First Faculty of Medicine, Charles University and General University Hospital in Prague, Prague, Czech Republic

**Keywords:** mtDNA, *MT-ND* genes, Complex I, Leigh syndrome, MELAS syndrome, MEGS, Mitochondria

## Abstract

**Background:**

Maternally inherited complex I deficiencies due to mutations in *MT-ND* genes represent a heterogeneous group of multisystem mitochondrial disorders (MD) with a unfavourable prognosis. The aim of the study was to characterize the impact of the mutations in *MT-ND* genes, including the novel m.13091 T > C variant, on the course of the disease, and to analyse the activities of respiratory chain complexes, the amount of protein subunits, and the mitochondrial energy-generating system (MEGS) in available muscle biopsies and cultivated fibroblasts.

**Methods:**

The respiratory chain complex activities were measured by spectrophotometry, MEGS were analysed using radiolabelled substrates, and protein amount by SDS-PAGE or BN-PAGE in muscle or fibroblasts.

**Results:**

In our cohort of 106 unrelated families carrying different mtDNA mutations, we found heteroplasmic mutations in the genes *MT-ND1*, *MT-ND3*, and *MT-ND5*, including the novel variant m.13091 T > C, in 13 patients with MD from 12 families. First symptoms developed between early childhood and adolescence and progressed to multisystem disease with a phenotype of Leigh or MELAS syndromes. MRI revealed bilateral symmetrical involvement of deep grey matter typical of Leigh syndrome in 6 children, cortical/white matter stroke-like lesions suggesting MELAS syndrome in 3 patients, and a combination of cortico-subcortical lesions and grey matter involvement in 4 patients. MEGS indicated mitochondrial disturbances in all available muscle samples, as well as a significantly decreased oxidation of [1-^14^C] pyruvate in fibroblasts. Spectrophotometric analyses revealed a low activity of complex I and/or complex I + III in all muscle samples except one, but the activities in fibroblasts were mostly normal. No correlation was found between complex I activities and mtDNA mutation load, but higher levels of heteroplasmy were generally found in more severely affected patients.

**Conclusions:**

Maternally inherited complex I deficiencies were found in 11% of families with mitochondrial diseases in our region. Six patients manifested with Leigh, three with MELAS. The remaining four patients presented with an overlap between these two syndromes. MEGS, especially the oxidation of [1-^14^C] pyruvate in fibroblasts might serve as a sensitive indicator of functional impairment due to *MT-ND* mutations. Early onset of the disease and higher level of mtDNA heteroplasmy were associated with a worse prognosis.

## Background

Disturbances of the respiratory chain complex I (CI, NADH:coenzyme Q oxidoreductase, EC 1.6.5.3) represent the most common cause of multisystem mitochondrial disorders (MD), accounting for nearly one-third of patients [1]. CI consists of 45 protein subunits with different functions necessary for enzyme assembly, stabilization, and regulation [2], encoded by genes in nuclear or mitochondrial DNA (mtDNA). Many mutations in these genes have already been described including in 22 genes for structural proteins and 11 genes for non-structural proteins encoded by nuclear DNA (Additional file 1: Table S1), and in all 7 mtDNA genes (*MT-ND1–6* and *MT-ND4L)* for structural subunits of CI. With respect to maternally inherited mutations, 28 different mutations sites have been confirmed, and another 113 sites in *MT-ND* genes have been published (www.mitomap.org).

Clinically, CI deficiency represents a heterogeneous group of MDs with an early, neonatal onset of fatal lactic acidosis; infantile onset of progressive mitochondrial encephalopathy with Leigh syndrome (LS); onset of Mitochondrial Encephalopathy, Lactic Acidosis and Stroke-like episodes (MELAS) syndrome during childhood, or adult-onset encephalomyopathic syndromes with various severities. Leber Hereditary Optic Neuropathy (LHON) syndrome with acute or subacute loss of vision usually starts during the second or third decade of life. In addition, several studies have also documented the LHON/MELAS overlap syndromes [3–5].

The prognosis in patients with maternally inherited complex I deficiencies is unfavourable and hardly predictable. In the cohort of 13 patients with MD due to 8 different mtDNA mutations in *MT-ND1*, *MT-ND3* and *MT-ND5* genes, including one novel variant m.13091 T > C in *MT-ND5* gene, we characterized the impact of the mutations in *MT-ND* genes on the course of the disease and we analysed their biochemical consequences in available muscle biopsies and cultivated skin fibroblasts.

## Material and methods

### Patients

Our laboratory serves as the diagnostic centre for MD in the Czech Republic, a country with 10.5 million inhabitants. During the last 25 years, different maternally inherited mtDNA mutations have been diagnosed in 106 unrelated families and sporadic large-scale deletions in mtDNA in 25 patients with Kearns-Sayre/Pearson syndromes. mtDNA mutations in genes for structural subunits of CI were present in 47 families, including 12 families with 13 patients with multisystem diseases due to heteroplasmic mtDNA mutations in *MT-ND1*, *MT-ND3* and *MT-ND5*, and 35 families with CI deficiency and LHON syndrome with tissue-specific optic nerve involvement due to homoplasmic mutations in m.3460G > A in *MT-ND1*, m.11777G > A in *MT-ND4* and m.14484 T > C in *MT-ND6.*

### Methods

#### Analysis of mtDNA

In patients 1–8 and 10, total genomic DNA isolated from muscle biopsy or cultivated skin fibroblasts (P2) and mtDNA (NC_012920) was sequenced as described previously [6]. In patients 9 and 11–13, the mtDNA mutation m.13513G > A was detected by PCR-RFLP using mismatch primers (F: 5′-GTTTGCGGTTTCGATGATGTGAT-3′; R: 5′- AACCATACC-TCTCACTTCAACCTCCC-3′) and Bsp143I endonuclease (Thermo Fisher Scientific, Waltham, Massachusetts, USA). Levels of heteroplasmy in available tissues were determined by mutation-specific PCR-RFLP analyses. Digested products were separated on an Agilent 2100 Bioanalyzer using High Sensitivity DNA kits. For each sample, the intensities of the individual restriction fragments were determined using Agilent 2100 Expert Software (Agilent Technologies, Santa Clara, California, USA). The level of heteroplasmy was calculated as the percentage of fragment intensity corresponding to the mutated mtDNA molecule. The detection limit of this method is 3%.

#### Isolation of mitochondria

Samples obtained by muscle biopsy were transported on ice (at 4 °C) and mitochondria were isolated immediately according to standard differential centrifugation procedures [7] in a buffer containing 150 mM KCl, 50 mM Tris/HCl, 2 mM EDTA and 2 μg/ml aprotinin (pH 7.5) at 4 °C. The homogenate was centrifuged for 10 min at 4 °C and 600 g, the supernatant was filtered through a 100 μm nylon membrane, and mitochondria were obtained by centrifugation for 10 min at 4 °C and 10,000 g. The mitochondrial pellet was washed and resuspended to a final protein concentration of 20–25 mg/ml [8].

#### Spectrophotometry

The activities of respiratory chain complexes (complex I − NADH:coenzyme Q oxidoreductase, CI, EC 1.6.5.3; complex I + III − NADH:cytochrome *c* oxidoreductase, CI + III; complex II − succinate:coenzyme Q oxidoreductase, CII, EC 1.3.5.1; complex II + III − succinate:cytochrome *c* oxidoreductase, CII + III; complex III − coenzyme Q:cytochrome *c* oxidoreductase, CIII, EC 7.1.1.8; complex IV − cytochrome *c* oxidase, CIV, EC 1.9.3.1) were measured according to [9]. The activity of citrate synthase (CS, EC 2.3.3.1), serving as the control enzyme to avoid assay variability, was measured according to [10]. Protein concentrations were measured by the Lowry method [11].

#### Electrophoresis

Blue Native Polyacrylamide Gel Electrophoresis (BN-PAGE) separation [12] of mitochondrial membrane complexes on polyacrylamide 4–14% or 6–15% (w/v) gradient gels (MiniProtean® 3 System; Bio-Rad, Hercules, California, USA), followed by immunoblot analysis was used to analyse the steady-state levels of oxidative phosphorylation system complexes [13]. Primary detection of BN-PAGE blots was performed using mouse monoclonal antibodies against the CI subunit NDUFA9 (1:2000), complex II subunit SDH70 protein (1: 6666), complex III subunit Core 2 (1:20000), complex IV subunit COX1 (1:3000) and ATP synthase subunit alpha (1:2000) (Abcam, Cambridge, UK). Sodium Dodecyl Sulfate Polyacrylamide Gel Electrophoresis **(**SDS-PAGE) was performed on 12% (w/v) polyacrylamide minigels (MiniProtean® 3 System) according to Schägger and von Jagow [14]. Primary antibodies against the CI subunits ND5 (1:2000), NDUFA9 (1:4000), and NDUFB6 (1:3000); complex II subunit SDH70 (1:20000); complex III subunits Core 1 (1:2000) and Core 2 (1:40000); complex IV subunit COX2 (1:10000) (all from Abcam) and control cytosol marker β-tubulin (1:4000; Sigma, St. Louis, Missouri, USA) were used for the detection of SDS-PAGE membranes. The immunoblots were detected with peroxidase-conjugated secondary antibodies and SuperSignal West Femto Maximum Sensitivity Substrate (Thermo Fisher Scientific) using G:Box (Syngene, Cambridge, UK) and analysed by Quantity One software (Bio-Rad).

#### MEGS analysis

The analysis of the mitochondrial energy-generating system (MEGS) was performed in 10 incubations containing ^14^C-labelled pyruvate, malate and succinate, donors and acceptors of Acetyl-CoA and inhibitors of TCA cycle, according to Janssen [15]. Briefly, each incubation contains the buffer for MEGS (30 mM KH_2_PO_4_ pH 7.4; 75 mM KCl; 8 mM Tris; 1.6 mM EDTA; 5 mM MgCl_2_; 0.2 mM p1,p5-di (adenosine-5′) pentaphosphate (myo-adenylate kinase inhibitor), and where indicated, 2 nM ADP; 1 mM pyruvate; 1 mM malate; 1 mM succinate (all from Sigma); with combinations of [1-^14^C] pyruvate (PerkinElmer, Waltham, Massachusetts, USA); [U-^14^C] malate (PerkinElmer) and [1,4-^14^C] succinate (Moravek Biochemicals, Brea, California, USA); 5 mM L-carnitine; 2 mM acetyl-D,L-carnitine; 2 mM sodium arsenite; 5 mM malonate; 2 μM CCCP and 40 μM atractyloside (all from Sigma). The composition of individual incubations is summarized in Additional file 1: Table S2. Incubations were performed in a shaking water bath at 37 °C in 0.2 ml glass incubation vials with caps and rubber septa. The measurement was started by adding 5 μl of whole cell lysate of fibroblasts and stopped after 20 min by 50 μl 3 M HClO_4_. The CO_2_ produced was trapped on a filter paper (saturated by 1 M NaOH) in each cap for 1 h at 4 °C; the filter paper was then transferred to a scintillation vial with 3 ml BCS solution (Amersham, Little Chalfont, UK) and ^14^CO_2_ was counted after 24 h in the Beckman Coulter LS6500 (Beckman Coulter, Brea, California, USA). Due to repeated measurements, the data related to protein amounts were analysed using a logistic regression model with mixed effects, where the studied reaction was perceived as a disease predictor and patient subjects or controls as a random effect. *P*-values less than 0.05 were considered statistically significant. Analysis was performed in R 3.5.1 statistical package, R Core Team (2018).

## Results

Multisystem MDs caused by mtDNA mutations in genes for structural subunits of CI were diagnosed in 13 patients from 12 families (P1 and P2 were cousins; their mothers are sisters), representing 11% of families with reported mtDNA mutations in our geographical region. Altogether, 8 different heteroplasmic mtDNA mutations in the genes *MT-ND1*, *MT-ND3*, and *MT-ND5* were found, including one novel variant m.13091 T > C (p.Met252Thr) in *MT-ND5.* The mutations and the levels of mtDNA heteroplasmy in muscle biopsies, cultivated fibroblasts, and other tissues are shown in Table 1. The most frequent were mutations in *MT-ND5* (69%). The same mtDNA mutations as in patients were also found in six mothers (of P1, P4, P5, P8, P10, and P11) and in two sisters (of P4 and P10) at least in one out of three or four examined tissues (blood, urinary sediment, buccal smear or hair follicles). All were asymptomatic, except the mother of P8 who had repeated attacks of migraine. No mutations were detected in the other six mothers; the mother of P2 was not analysed.

**Table 1:** Clinical and laboratory data in 13 patients with complex I deficiency

Patient	1	2	3	4	5	6	7	8	9	10	11	12	13
mtDNA gene	*MT-ND1*			*MT-ND3*	*MT-ND5*								
mutation	m. 3697 G > A		m. 3946 G > A	m. 10158 T > C	m. 12706 T > C	m. 13042G > A	m. 13046 T > C	m. 13091 T > C	m. 13513G > A				
muscle mtDNA heteroplasmy [%]	93	np	53	95	83	96	70	61	67	48	97	np	np
fibroblasts mtDNA heteroplasmy [%]	81	79	np	85	np	65	43	np	65	40	4	np	np
blood mtDNA heteroplasmy [%]	93	96	np	90	3	96	27	4	58	44	35	21	64
hair follicles mtDNA heteroplasmy [%]	93	np	np	94	np	90	44	12	np	66	86	9	4
urinary sediment mtDNA heteroplasmy [%]	96	np	np	np	47	89	71	52	np	81	92	74	80
buccal smear mtDNA heteroplasmy [%]	93	np	np	92	np	93	34	30	np	55	68	5	71
median for all tissues	93	88	53	92	47	92	44	30	65	52	77	15	68
age at onset (week, months, years)	1 w	1 w	9 y	4 m	17 y	6 m	12 y	10 y	1 m	6 y	10 y	10 y	5 m
first symptom	hypotony	hypotony	stroke-like episode	hypotony	Wernicke aphasia	hypotony	optic neuuropathy	migraine	hypotony	optic neuropathy	stroke-like episode	hearing loss	nystagmus
failure to thrive	**+**	**–**	**+**	**+**	**–**	**–**	**–**	**–**	**+**	**–**	**–**	**+**	**–**
initial hypotony/ later spasticity	**+/+**	**+/+**	**+/+**	**+/+**	**−/−**	**+/+**	**−/−**	**−/−**	**+/+**	**−/−**	**−/−**	**+/−**	**+/−**
delayed motor development	**+**	**+**	**+**	**+**	**–**	**+**	**–**	**–**	**+**		**–**		**–**
cerebellar symptoms	**+**	**–**	**+**	**+**	**+**	**+**	**–**	**–**	**+**	**+**	**+**	**+**	**–**
strabismus	**+**	**+**	**+**	**+**	**–**	**+**	**–**			**+**	**–**	**+**	**+**
epilepsy	**+**	**–**	**+**	**+**	**+**	**–**	**–**	**+**	**+**	**+**	**+**	**+**	**–**
migraine	**–**			**–**	**+**	**–**	**+**	**+**	**–**	**+**	**+**	**+**	**–**
optic atrophy	**–**			**–**	**–**	**+**	**+**	**+**	**–**	**+**	**+**	**+**	**–**
ptosis	**–**			**–**	**–**	**+**	**–**	**–**	**+**	**+**	**+**	**+**	**+**
CPEO	**–**		**+**	**–**	**–**	**+**	**–**	**–**	**–**	**+**	**–**	**+**	**+**
visual impairment	**+**	**+**		**–**		**+**		**+/−**	**+**	**+**	**–**	**+**	**+**
hearing loss	**+**		**+**	**+**	**+**	**–**	**+**	**–**	**+**	**+**	**+**	**+**	**–**
peripheral neuropathy	**–**			**+**			**–**	**–**	**–**	**–**	**+**		**–**
mental insufficiency	**+**	**+**	**+**	**+**		**–**	**–**	**–**	**+**	**+**	**–**	**+**	**–**
psychiatric disturbances			**+**		**+**	**–**	**+**	**–**		**+**	**+**		**–**
present age (years)	died at 7	died at 1.8	31	died at 1.3	died at 42	6.5	17	31	died at 3.3	20.5	25	22	6.5
creatine kinase [controls < 2.5 ukat/l]	1–2.4	0.8	1	0.66	2–3.8	np	1–190	1–1.7	0.6	np	0.6–2	1.5–14	2–10
blood-lactate [controls < 2.3 mmol/l]	2–6	3–6	2.7–4.4	3–7	3–8	2.8–5	1.5–13	1.2–2.6	2–3.4	8.6	2–5	2–5	2.4–4
CSF-lactate [controls < 2.1 mmol/l]	3	4	7	5.6	4.5	3.9	4.3	4.2	3.6	14	6.2	np	4.3
age at MRI (months, years)	2 y	7 m	14 y		36 y	20 m	17 y	32 y	34 m	9 y	25y	18 y	3 y
MRI – bilateral deep gray matter lesions^a^	+	+	+	+	–	+	+	–	+	+	–	+	+
MRI –stroke-like lesions^b^	+	–	+	–	+	–	+	+	–	–	+	+	–
MRI –periventricular atrophy	++	+	++	–	++	–	–	+	+	–	+	+	–

*Abbreviations*: *CPEO* chronic progressive external ophthalmoplegia, *PM* psychomotor, *CSF* cerebrospinal fluid (CSF lactate in P10 analysed at stroke-like episode); np - not performed

^a^Compatible with Leigh syndrome (see Fig. 1), ^b^compatible with MELAS syndrome (see Fig. 1)

### Clinical data

Clinical data are outlined in Table 1. The onset of the disease varied from the neonatal period to 17 years, and three main phenotypes were observed: 1) Six children developed LS (P1, P2, P4, P6, P9, and P13) associated with a worse prognosis, because four of them died between the age of 16 months and 7 years. Two surviving children with LS (P6 and P13) are 6.5-year-old girls. 2) Five children developed MELAS syndrome (P3, P5, P8, P11, and P12) with a history of stroke-like episodes, and 3) the last two children (P7 and P10) had LHON-like onset of the disease with optic neuropathy at the age of 12 years and 6 years, respectively. Nevertheless, both of them later developed multisystem symptoms and transitioned to MELAS syndrome or LHON/MELAS overlap syndrome. In addition, P9 and P11 also had MELAS syndrome with some degree of optic neuropathy with alterations in Best Corrected Visual Acuity (BCVA), and P12 had limited size of the optic discs. Hypertrophic cardiomyopathy (HCMP) with Wolf-Parkinson-White (WPW) syndrome was present in P9 and P13. Most patients also exhibited intermittent or permanent increases of lactate in the blood (B-lactate 1.2–13 mmol/l, controls < 2.3 mmol/l) and the cerebrospinal fluid (CSF-lactate 3–14 mmol/l, controls < 2.3 mmol/l).

**Fig. 1:**
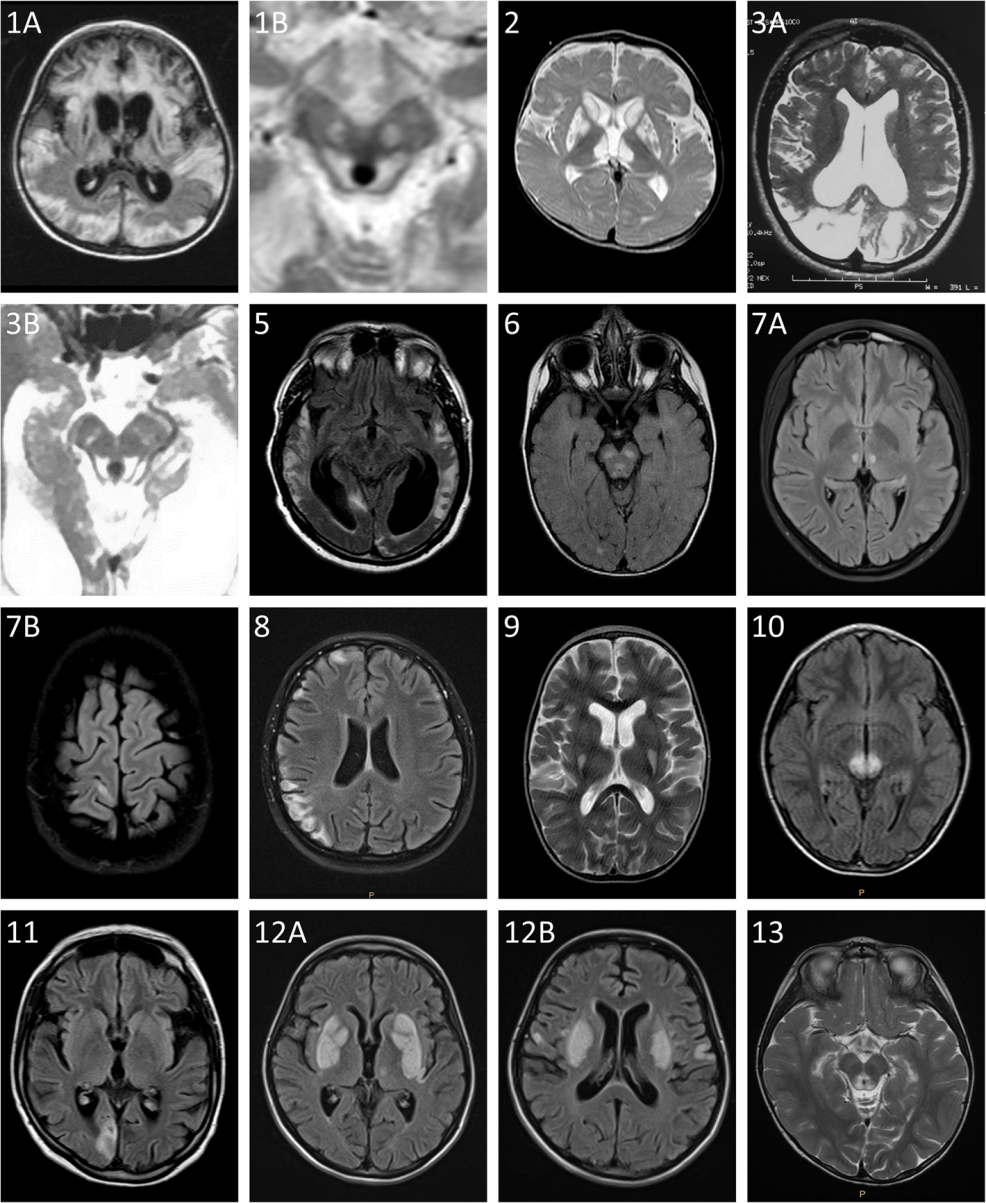
MRI of the brain in P1–P3 and P5–P12 with complex I deficiency. Signal changes in basal ganglia or brainstem characteristic for Leigh syndrome are present in patients P2, P6, P9, P10 and P13. Signal changes in the cortex and white matter of the hemispheres or cerebellum with stroke-like lesion are present in patients P5, P8, P11, and the combination of both changes are visible in patients P1, P3, P7 and P12. Moderate to severe periventricular atrophy was found in patients P1, P3 and P5.

Brain MRI was performed in all patients, albeit at different ages (Table 1). In 6 of them (P2, P4, P6, P9, P10, and P13), symmetric signal changes in deep grey matter structures, including the basal ganglia and the brainstem, characteristic for LS, were found. Signal changes in the cortex and white matter of the hemispheres and the cerebellum resembling stroke-like lesions of MELAS syndrome were found in 3 patients (P5, P8, and P11), and the combination of both changes typical for LS and stroke-like lesions was present in 4 patients (P1, P3, P7, and P12). In addition, moderate to severe periventricular atrophy was found in three patients (P1, P3, and P5). Brain MRI images of all patients, except P4, are shown in Fig. 1.

### Biochemical measurement

In isolated muscle mitochondria, the activity of CI/CS was decreased or borderline low in 8 of 10 analysed patients (P3–P8, P10, and P11), and the activity of the respiratory chain CI + III/CS was low in 9 of 10 analysed patients (P3–P11). Activities of other oxidative phosphorylation (OXPHOS) complexes were altered in some patients. CII + III/CS was increased in 3 patients, CIII/CS was elevated in 3 patients, and CIV/CS was altered in 4 patients (3 increased, 1 decreased), see Table 2. We observed no significant correlation between enzymatic activities and the heteroplasmy of mtDNA mutations. The activities of CI in cultivated fibroblasts were within the reference range (data not shown).

**Table 2:** The activities of respiratory chain complexes in isolated muscle mitochondria

Patient	Individual respiratory chain complexes activities in isolated muscle mitochondria											
	CI/CS		CI + III/CS		CII/CS		CII + III/CS		CIII/ CS		CIV/CS	
	patient	age related control range	patient	age related control range	patient	age related control range	patient	age related control range	patient	age related control range	patient	age related control range
1	0.50	0.45–1.05	0.18	0.17–0.31	0.09	0.07–0.27	0.33	0.17–0.47	0.43	0.27–0.85	1.59	1.1–2.22
3	**0.13**	0.18–0.38	**0.08**	0.18–0.37	**0.04**	0.05–0.11	0.24	0.17–0.32	0.67	0.46–0.88	1.21	0.66–2.25
4	**0.24**	0.45–1.05	**0.06**	0.17–0.31	0.09	0.07–0.27	0.34	0.17–0.47	**0.95**	0.27–0.85	**0.97**	1.1–2.22
5	**0.11**	0.15–0.41	**0.01**	0.13–0.25	0.05	0.05–0.11	0.23	0.15–0.27	**0.72**	0.3–0.56	1.5	0.66–2.25
6	**0.06**	0.45–1.05	**0.08**	0.17–0.31	0.07	0.07–0.27	0.26	0.17–0.47	0.72	0.27–0.85	1.12	1.1–2.22
7	**0.08**	0.18–0.38	**0.06**	0.18–0.37	0.07	0.05–0.11	**0.38**	0.17–0.32	0.74	0.46–0.88	**1.11**	1.16–2.13
8	0.16	0.15–0.41	**0.05**	0.13–0.25	0.06	0.05–0.11	**0.44**	0.15–0.27	**1.7**	0.3–0.56	1.12	0.66–2.25
9	0.28	0.18–0.38	**0.03**	0.18–0.37	0.09	0.05–0.11	**0.38**	0.17–0.32	0.66	0.46–0.88	1.27	1.16–2.13
10	**0.11**	0.18–0.38	**0.08**	0.18–0.37	0.05	0.05–0.11	0.30	0.17–0.32	0.57	0.46–0.88	**1.7**	1.16–2.13
11	**0.07**	0.18–0.38	**0.03**	0.18–0.37	**0.04**	0.05–0.11	0.19	0.17–0.32	0.52	0.46–0.88	**0.96**	1.16–2.13

The reference ranges for individual patients according to age of patients are displayed in next column. Controls are constituted in the three major groups (0–2 years old; 3–18 years old and adults). Alterations in patient’s complexes activities are shown in bold. *Abbreviations*: *CI* complex I, *NADH* coenzyme Q reductase; *CI + III* complex I + III, *NADH* cytochrome c reductase; *CII* complex II, succinate:coenzyme Q reductase; *CII + III* complex II + III, succinate:cytochrome *c* oxidoreductase; *CIII* complex III, coenzyme Q:cytochrome *c* oxidoreductase; *CIV* complex IV, cytochrome *c* oxidase; *CS* citrate synthase

**Fig. 2:**
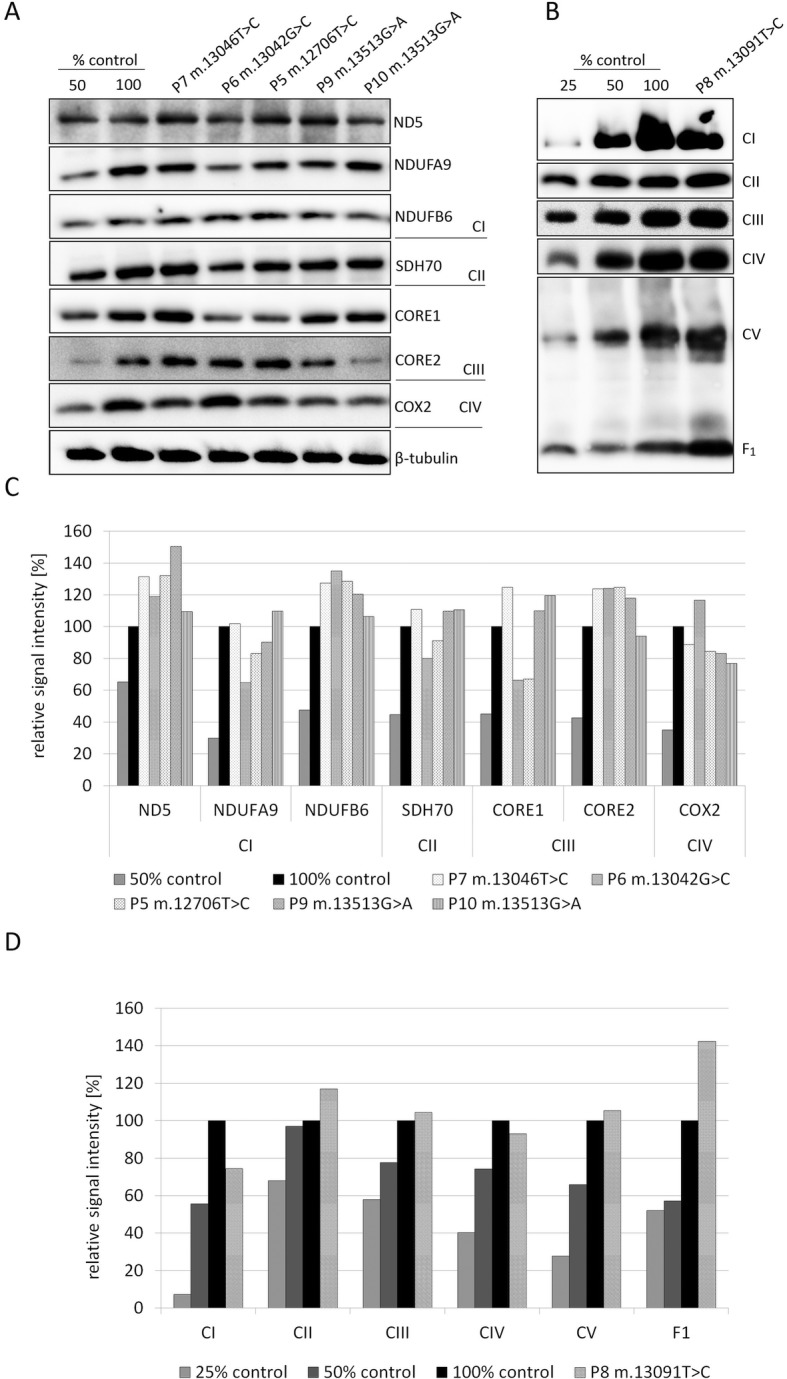
Protein analysis in six patients with heteroplasmic mutations in MT-ND5 gene. **a** Comparison of steady-state levels of several OXPHOS-related proteins in P5, P6, P7, P9 and P10 in fibroblasts using SDS-PAGE/WB. As a control was used primary dermal fibroblasts (ATCC® PCS-201-010™), 50 and 100% demonstrate loading dose of protein amount. **b** BN-PAGE/WB of OXPHOS complexes in isolated mitochondria from the muscle of P8. As a control was used human muscle mitochondria from heathy adult, 25; 50 and 100% demonstrate loading dose of protein amount. **c** The quantification of western blot signals from A by densitometric analysis. Relative signals intensity of individual OXPHOS antibodies were normalized to intensity of loading control β-tubulin. **d** The quantification of western blot signals from B by densitometric analysis

SDS-PAGE/Western blots (WB) in cultivated fibroblasts of patients with *MT-ND5* mutations revealed slightly increased amounts of the ND5 subunit (Fig. 2a and c) in three patients (P5, P7 and P9). We noted mild alterations in the amount of selected OXPHOS protein subunits in comparison to age-related controls, but they were not uniform across the group of patients.

**Fig. 3:**
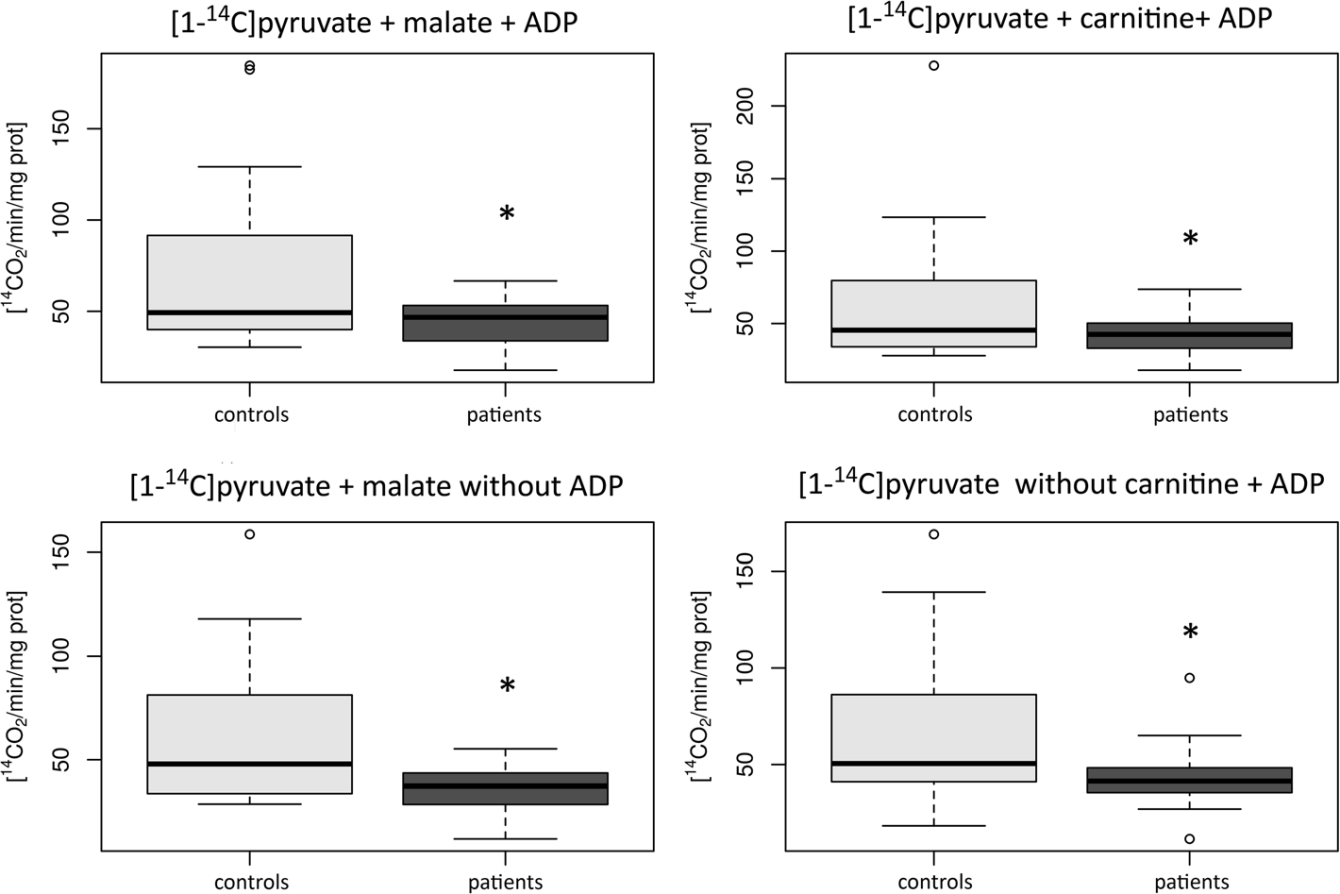
Oxidation rate of 4 different MEGS incubations containing [1-^14^C] pyruvate in cultivated skin fibroblasts. Patient group consists of 8 patients (P1, P2, P4, P6, P7, and P9–P11) with complex I deficiency and heteroplasmic mutations in *MT-ND1, MT-ND3* and *MT-ND5* genes. Control group consists of 10 age-related controls. In the group of patients, oxidation rate of all four displayed MEGS incubations are significantly decreased (*p < 0.05). Open circles displayed suspected outliers

The MEGS analysis in cultivated skin fibroblasts from 8 patients (P1, P2, P4, P6, P7, and P9–P11) revealed decreased oxidation rates in 4 incubations containing [1-^14^C] pyruvate (Fig. 3). Oxidation rates of the other 2 incubations containing [1-^14^C] pyruvate and incubations containing [U-^14^C] malate and [1,4-^14^C] succinate were similar to controls. MEGS analyses were also performed in muscle postnuclear homogenate in 4 patients (P4, P5, P7 and P9). In all samples, MEGS revealed disruption at the level of the respiratory chain (data not shown).

### The novel variant

The novel variant m.13091 T > C (p.Met252Thr) in the *MT-ND5* gene was found in P8 (Table 1). She was born at term to healthy parents and was asymptomatic until the age of 10 years, when frequent attacks of migraine started. At the age of 26 years, she developed repeated stroke-like episodes with secondary epilepsy. She has had no ptosis or chronic ophthalmoplegia. Cardiologic examination and audiometry were uneventful, but the CSF-lactate was elevated (4.2 mmol/l, controls < 2.3 mmol/l) and both optic nerves appeared pale on ophthalmoscopy. Perimetric investigation revealed multiple bilateral scotomas, and visual evoked potentials showed reduced amplitudes with prolonged P100 wave latencies. Ophthalmological assessment revealed bilateral pallor of the optic discs and decreased retinal nerve fibre layer thickness in all four quadrants (OCT Spectralis, Heidelberg Engineering, Germany). Nevertheless, the patient’s visual acuity was normal, bilaterally (Best Corrected Visual Acuity 1.0). Muscle biopsy revealed a mild focal subsarcolemmal accumulation of SDH product (3%) with a decreased activity of CI + III in isolated mitochondria (Table 2), while the activities of CI and other respiratory chain complexes were within the control range, except for the increased activity of respiratory chain CII + III (probably as the compensatory impact of CI + III deficiency). BN-PAGE revealed decreased amounts of CI in muscle to approx. 74%; the amount of other OXPHOS complexes remained unchanged (Fig. 2b and d) except ATP synthase, where we observed a mildly increased amount of free F_1_ domain in comparison to control. Both the patient’s mother and sister suffer from migraines. However, in the patient’s mother, only a 12% mutational load of m.13091 T > C was present in urine, while blood, buccal smears and hair follicles were negative. The mutational load in samples from the patient’s sister was below the detection limit of the method.

## Discussion

Maternally inherited mutations in *MT-ND*, genes for structural subunits of CI, may be found in > 30% of patients with isolated CI deficiency [16, 17]. Most of them have LHON syndrome with isolated optic neuropathy due to the prevalent mutations m.11777G > A in *MT-ND4*, m.3460G > A in *MT-ND1*, or m.14484 T > C in *MT-ND6*. Multisystem mitochondrial disorders are less frequent. The *MT-ND5* gene appears to be clearly a hotspot for disease-causing mutations [18], and the m.13513G > A mutation is one of the most common [19]. Although the *MT-ND5* gene is the largest of the mtDNA-encoded genes for CI (1812 bp), this alone does not explain the increased number of mutations in this gene compared with other mitochondrial genes [20]. Still, it corresponds well with the results of molecular analyses in our families with multisystem MD, because most of them have mutations in the *MT-ND5* gene, including one girl with the novel heteroplasmic mutation m.13091 T > C. Similarly to other reports [19, 21, 22] also in our group of patients, the most frequent mutation in the *MT-ND5* gene was m.13513G > A. The second most common group of mutations in our subjects were mutations in the *MT-ND1* gene, found in three patients. One of the mutations, m.3946G > A, was the first within *MT-ND* genes associated with the MELAS syndrome phenotype [23].

The phenotype in patients with CI deficiency may have overlapping features of different mitochondrial syndromes. Our patients manifested, most frequently during childhood or adolescence, with LS with progressive motor and subsequent mental deterioration, or with MELAS syndrome, lactic acidosis and attacks of stroke-like episodes. Less frequent was the onset of optic neuropathy followed with multisystem symptoms representing LHON/MELAS overlap syndrome. Hypertrophic CMP with or without WPW syndrome is not rare in patients with the mutation m.13513G > A [19, 21, 22, 24, 25]; we observed it in 2 patients. Severe rhabdomyolysis was observed only once, in P7.

Clinical symptoms in P8 with MELAS syndrome and the novel heteroplasmic variant m.13091 T > C in *MT-ND5* started with attacks of migraine at the age of 10 years and stroke-like episodes at the age of 26 years. In addition, she developed myopathy, optic neuropathy and secondary epilepsy. This course of the disease is compatible with our observation that any symptom from the broad phenotypic spectrum of MELAS syndrome may come first and stay isolated for a long period of time [26]**.**

Three patients with mutations in *MT-ND1* also developed an acute encephalitic episode characterized by an acute qualitative alteration of consciousness with hyporesponsivity and profound hypotonia. This may have been caused by selective neuronal impairment due to energetic deprivation, leading to reversible ischaemic damage that lacks evidence of decreased tissue perfusion [27]. Some patients may recover to a normal or nearly normal condition as before the event [1, 19, 22]. Of some interest is the Wernicke’s aphasia in P5, with mutation m.12706 T > C in *MT-ND5*, which was characterized by a difficulty of understanding written and spoken language. The cause of Wernicke’s aphasia is usually an ischaemic stroke affecting the Wernicke’s area in the posterior temporal lobe of the dominant hemisphere perfused by branches of the middle cerebral artery [28]. MRI of the brain revealed a typical ischemic hyperintensity in the left temporoparietal region. In contrast, MELAS lesions do not follow vascular territories or a border zone and the cerebral angiography fails to demonstrate any steno-occlusive lesions [29]. As far as we know, only one mitochondrial patient with mtDNA mutation m.3243G > A has previously been described with Wernicke’s aphasia [30].

Vascular dysfunction seems to play an important role in the pathogenesis of MD. However, the affected areas, as observed via neuroimaging, do not always correspond to classic regional vascular distributions; therefore, they are called “stroke-like” lesions. Additionally, other pathophysiological mechanisms may contribute to or be responsible for the development of stroke-like episodes. One of them is a generalized cytopathy caused by critical energy deprivation in neurons and/or glia. The cytotoxicity may lead to either temporary or permanent lesions depending on the level of energetic failure [31, 32]. It was also hypothesized that the abnormal mitochondria in the endothelium may disturb the blood–brain barrier, resulting in altered ion homeostasis, hyperexcitability and focal epileptic activity [33]. Using MRI and ^1^H magnetic resonance spectroscopy, different white and grey matter lesions may be found in the central nervous system of patients with CI deficiency. Caudate lesions were more common in patients with mtDNA mutations, as opposed to patients with nuclear mutations [34].

LS is characterized by symmetric involvement of the deep grey matter. The most commonly affected structures are the substantia nigra, putamen, nucleus dentatus and the brainstem; however, the thalamus, cerebellum and grey matter of the spinal cord may also be involved, and lesions in white matter structures were also described [35]. The deep grey matter lesions may result from several factors, including ATP depletion, gliosis, high lactate and excessive reactive oxygen species production [36]. In patients with MELAS syndrome, stroke-like lesions with the involvement of grey matter and subcortical white matter typically exhibit a “shifting spread” (appearance, disappearance and re-appearance). These lesions are characterized by asymmetric signal changes crossing vascular territories and an abnormally prominent lactate peak [37, 38]. Some patients also have small lesions in the deep grey matter (either less symmetrical than in LS or unilateral) and calcifications in basal ganglia.

In our patients with maternally inherited CI deficiency, MRI revealed both the involvement of deep grey matter structures and stroke-like lesions similar to other reports [4]. We also demonstrated the coexistence of LS and stroke-like lesions in 4 of the 13 patients, suggesting that an overlap between the LS and MELAS phenotypes is not rare in patients with maternally inherited CI deficiency. Moderate cortical or periventricular atrophy was present in 4 of 13 patients, but these atrophies have been shown to be non-specific in patients with mitochondrial diseases [34].

Grey matter has been estimated to consume approximately 2.5-fold more ATP than white matter [39]. It is therefore not surprising that the majority of MDs present with predominant involvement of grey matter that may extend to secondary white matter degeneration [36], similar to what was observed in the subcortical regions in some of our patients. It was hypothesized that LS is typical in patients with very high levels of mtDNA mutation heteroplasmy [36]. However, this was only partly supported by our study, as some of our patients with high levels of heteroplasmy manifested stroke-like phenotypes. In fact, isolated LS and LS/MELAS overlap syndromes were present even in some patients with lower levels of heteroplasmy.

### Prognosis

The prognosis in most patients with multisystem MD and CI deficiency is not good. Similarly to other reports [1, 19, 22, 40], our patients with higher levels of heteroplasmy (expressed as the median of heteroplasmy from all analysed tissues) had poorer prognosis with LS phenotype and an earlier onset of the disease, whereas patients with lower heteroplasmy levels developed milder MELAS or LHON/MELAS phenotypes with a later onset [19, 20, 41, 42]. On the other hand, LS was also described in some patients with low levels of heteroplasmy [21, 43, 44]. It was shown in patients with MELAS syndrome, including m.13513G > A mutation, that L-arginine infusion during the acute phase of the stroke-like episode may reduce acute symptoms, and oral supplementation with L-arginine and/or L-citruline may prevent further stroke-like episodes, with arginine acting as a nitric oxide donor, reversing the vasospasm [45]. A recent study at the basic research level has shown that in the induced pluripotent stem cells (iPSCs) with m.13513G > A, it is possible to decrease the level of heteroplasmy in iPSCs using transcription activator-like effector nucleases (TALENs) [46].

### Biochemical findings

The activities of CI and CI + III in isolated muscle mitochondria were decreased in most patients. Results of our study suggest that CI + III activity in muscle biopsy normalized to CS activity (serving as the control enzyme) is a good biochemical indicator for CI deficiency. This may be due to the CI assay measuring only the redox activity of the enzyme, which takes place within the peripheral arm, while mutations in the membrane arm subunits (which includes all seven mtDNA-encoded subunits [47]), may theoretically result in ostensibly normal enzymatic activity [1]. In 4/10 patients, CII + III activity was elevated, probably as a compensatory effect of CI deficiency. A similar effect was described in patients with multiple system atrophy with altered biosynthesis of the electron carrier CoQ10 as a consequence of a mutation in the *COQ2* gene [48]. The increase of CI activity that they observed could indicate a compensatory mechanism in response to downstream reduction in CII + III activity in cerebellar and occipital white matter [48]. In comparison to spectrophotometric analyses of muscle biopsies, similar measurements in cultivated fibroblasts for the diagnosis of CI deficiency in most of our patients were less predictive. In addition, no correlation was found between the activities and amounts of CI and CI + III in the muscle biopsies or cultivated fibroblasts, and the levels of heteroplasmic mutations in *MT-ND1*, *MT-ND3* and *MT-ND5* genes, similar to several other reports [19–21, 44]. Contrarily, a correlation between the level of heteroplasmy and the level of residual CI activity was described in cybrids derived from patients with mutations m.3481G > A in *MT-ND1,* m.10158 T > C and m.10191 T > C in *MT-ND3* and m.13063G > A in *MT-ND5* [48, 49]. However, the variable expression may be caused by different nuclear backgrounds, mtDNA haplotypes, environmental factors or ageing [43].

Recently, Kopinski et al. [50] showed how the level of mtDNA heteroplasmy changed the nuclear epigenome through metabolites, in cybrid models with an altered level of m.3243A > G encoded tRNA^Leu^ in the same nuclear background. They showed changes in nuclear gene expression via histone modification, which is modulated by the level of mitochondrially generated metabolites acetyl-CoA and α-ketoglutarate. Levels of those metabolites correlate with histone modifications, which differ across the different levels of heteroplasmy. They also revealed that mtDNA heteroplasmy affects mitochondrial NAD^+^/NADH ratio, which correlates with nuclear histone acetylation. Meanwhile, nuclear NAD^+^/NADH ratio correlates with changes in nDNA and mtDNA transcription. Hence, mutations in mtDNA generate particular metabolites and epigenetic changes at different heteroplasmy levels. This could explain the phenotypic variability of mitochondrial disease [50].

Simultaneous analyses of spectrophotometric and MEGS measurements in muscle biopsies from patients with MDs have already been performed in several studies [15, 51–53]. MEGS analysis is a sensitive method for detection of OXPHOS deficiency and the deficiency of adenine nucleotide translocator or pyruvate dehydrogenase complex (PDHc) in muscle tissue [15, 52]. In our cohort, MEGS analysis revealed disturbances of OXPHOS in all 4 patients where muscle tissue was examined. Moreover, in fibroblasts from 8 patients, MEGS analysis revealed decreased oxidation rates in 4 out of 6 incubations containing [1-^14^C] pyruvate, whereas the oxidation rates of incubations containing [U-^14^C] malate or [1,4-^14^C] succinate remained within the control range. Similarly, diminished [2-^14^C] pyruvate oxidation was also described in 8 out of 11 fibroblast lines from patients with CI deficiency [54]. The disturbances of CI led to an increased NADH/NAD^+^ ratio, resulting in the inhibition of PDHc activity and thus slowing down of the pyruvate oxidation rate [55, 56]. Combined evaluation of all MEGS parameters makes it possible to clearly distinguish suspicion for complex I deficiency.

The results of our study suggest that MEGS analyses may serve as a good indicator for CI deficiency and may help to accelerate the diagnostic flow. Both analyses – MEGS and spectrophotometry, especially the activity of complex I + III – are sensitive methods for the recognition of CI deficiency due to *MT-ND* mutation in muscle biopsy. In fibroblasts, MEGS seems to be more sensitive. Combining several biochemical methods may improve our understanding of the impact of individual mutations of *MT-ND* genes on mitochondrial bioenergetics.

## Conclusions

Patients with multisystem MDs due to heteroplasmic mtDNA mutations resulting in isolated CI deficiency represent approximately 11% of all families with maternally inherited MD diagnosed in our geographical region. MRI of the brain revealed the presence of LS in 6 out of 13 patients (46%), MELAS syndrome in 3/13 (23%), and an overlap between both syndromes in 4/13 (31%). All four patients with mutations in *MT-ND1* or *MT-ND3* had LS, whereas patients with *MT-ND5* mutations presented equally often with LS or MELAS. MEGS, especially oxidation of [1-^14^C] pyruvate might serve as a sensitive indicator of functional impairment due to *MT-ND* mutations in fibroblasts. Early onset of the disease and higher level of mtDNA mutation heteroplasmy were associated with worse prognosis.

## Supplementary information

**Additional file 1 : Table S1.** Structural and Non-Structural Nuclear Genes for Complex I disorders. **Table S2.** Composition of individual MEGS incubations.

## Data Availability

The datasets used and/or analysed during the current study are available from the corresponding author on reasonable request.

## Abbreviations

BCVA: Best Corrected Visual Acuity
BN-PAGE: Blue Native Polyacrylamide Gel Electrophoresis
CI + III: Respiratory chain complex I + III (NADH:cytochrome *c* oxidoreductase)
CI: Respiratory chain complex I (NADH:coenzyme Q oxidoreductase)
CII + III: Respiratory chain complex II + III (succinate:cytochrome *c* oxidoreductase)
CII: Respiratory chain complex II (succinate:coenzyme Q oxidoreductase)
CIII: Respiratory chain complex III (coenzyme Q:cytochrome *c* oxidoreductase)
CIV: Respiratory chain complex IV (cytochrome *c* oxidase)
CS: Citrate synthase
CSF: Cerebrospinal fluid
HCMP: Hypertrophic cardiomyopathy
iPSCs: Induced Pluripotent Stem Cells
LHON: Leber Hereditary Optic Neuropathy
LS: Leigh syndrome, symmetric necrotic lesions in basal ganglia and/or in the brain stem
MD: Mitochondrial disorders
MEGS: Mitochondrial energy-generating system
MELAS: Mitochondrial Encephalopathy, Lactic Acidosis and Stroke-like episode
mtDNA: mitochondrial DNA
OXPHOS: Oxidative phosphorylation
PDHc: Pyruvate dehydrogenase complex
RNFL: Retinal nerve fiber layer
SDS-PAGE: Sodium dodecyl sulfate polyacrylamide gel electrophoresis
TALENs: Transcription activator-like effector nucleases
WB: Western blot
WPW: Wolf-Parkinson-White syndrome
