# Supplementary material for: Multisystem mitochondrial diseases due to mutations in mtDNA-encoded subunits of complex I
Source: BMC Pediatr. 2020 Jan 29;20:41. doi: 10.1186/s12887-020-1912-x (PMC6988306; doi:10.1186/s12887-020-1912-x)
Supplement: Supplementary file 1 — Additional file 1 : Table S1. Structural and Non-Structural Nuclear Genes for Complex I disorders. Table S2. Composition of individual MEGS incubations. [file 12887_2020_1912_MOESM1_ESM.docx]

Table S1: Structural and Non-Structural Nuclear Genes for Complex I disorders

|  | **structural genes** | **references** |
| --- | --- | --- |
| 1 | *NDUFS1* | (Bénit et al., 2001) |
| 2 | *NDUFS2* | (Loeffen et al., 2001) |
| 3 | *NDUFS3* | (Bénit et al., 2004) |
| 4 | *NDUFS4* | (van den Heuvel et al., 1998) |
| 5 | *NDUFS6* | (Kirby et al., 2004, p. 6; Spiegel et al., 2009, p. 6) |
| 6 | *NDUFS7* | (Smeitink and van den Heuvel, 1999) |
| 7 | *NDUFS8* | (Loeffen et al., 1998) |
| 8 | *NDUFB3* | (Calvo et al., 2012) |
| 9 | *NDUFB8* | (Piekutowska-Abramczuk et al., 2018) |
| 10 | *NDUFB9* | (Haack et al., 2012) |
| 11 | *NDUFB10* | (Friederich et al., 2017) |
| 12 | *NDUFB11* | (Kohda et al., 2016) |
| 13 | *NDUFV1* | (Smeitink and van den Heuvel, 1999) |
| 14 | *NDUFV2* | (Bénit et al., 2003) |
| 15 | *NDUFA1* | (Fernandez-Moreira et al., 2007; Potluri et al., 2009) |
| 16 | *NDUFA2* | (Hoefs et al., 2008) |
| 17 | *NDUFA6* | (Alston et al., 2018) |
| 18 | *NDUFA9* | (van den Bosch et al., 2012) |
| 19 | *NDUFA10* | (Hoefs et al., 2011) |
| 20 | *NDUFA11* | (Berger et al., 2008) |
| 21 | *NDUFA12* | (Ostergaard et al., 2011) |
| 22 | *NDUFA13* | (Angebault et al., 2015) |
|  | **non-structural genes** | **references** |
| 1 | *NDUFAF1* | (Dunning et al., 2007) |
| 2 | *NDUFAF2* | (Ogilvie et al., 2005) |
| 3 | *NDUFAF3* | (Saada et al., 2009) |
| 4 | *NDUFAF4* | (Saada et al., 2008) |
| 5 | *NDUFAF5* | (Gerards et al., 2010) |
| 6 | *NDUFAF6* | (Bianciardi et al., 2016) |
| 7 | *NUBPL* | (Calvo et al., 2010) |
| 8 | *FOXRED1* | (Calvo et al., 2010) |
| 9 | *ACAD9* | (Haack et al., 2010) |
| 10 | *TIMMDC1* | (Kremer et al., 2017) |
| 11 | *TMEM126B* | (Sánchez-Caballero et al., 2016) |

References:

Alston, C.L., Heidler, J., Dibley, M.G., Kremer, L.S., Taylor, L.S., Fratter, C., French, C.E., Glasgow, R.I.C., Feichtinger, R.G., Delon, I., Pagnamenta, A.T., Dolling, H., Lemonde, H., Aiton, N., Bjørnstad, A., Henneke, L., Gärtner, J., Thiele, H., Tauchmannova, K., Quaghebeur, G., Houstek, J., Sperl, W., Raymond, F.L., Prokisch, H., Mayr, J.A., McFarland, R., Poulton, J., Ryan, M.T., Wittig, I., Henneke, M., Taylor, R.W., 2018. Bi-allelic Mutations in NDUFA6 Establish Its Role in Early-Onset Isolated Mitochondrial Complex I Deficiency. Am. J. Hum. Genet. 103, 592–601. https://doi.org/10.1016/j.ajhg.2018.08.013

Angebault, C., Charif, M., Guegen, N., Piro-Megy, C., Mousson de Camaret, B., Procaccio, V., Guichet, P.-O., Hebrard, M., Manes, G., Leboucq, N., Rivier, F., Hamel, C.P., Lenaers, G., Roubertie, A., 2015. Mutation in NDUFA13/GRIM19 leads to early onset hypotonia, dyskinesia and sensorial deficiencies, and mitochondrial complex I instability. Hum. Mol. Genet. 24, 3948–3955. https://doi.org/10.1093/hmg/ddv133

Bénit, P., Beugnot, R., Chretien, D., Giurgea, I., De Lonlay-Debeney, P., Issartel, J.-P., Corral-Debrinski, M., Kerscher, S., Rustin, P., Rötig, A., Munnich, A., 2003. Mutant NDUFV2 subunit of mitochondrial complex I causes early onset hypertrophic cardiomyopathy and encephalopathy. Hum. Mutat. 21, 582–586. https://doi.org/10.1002/humu.10225

Bénit, P., Chretien, D., Kadhom, N., de Lonlay-Debeney, P., Cormier-Daire, V., Cabral, A., Peudenier, S., Rustin, P., Munnich, A., Rötig, A., 2001. Large-scale deletion and point mutations of the nuclear NDUFV1 and NDUFS1 genes in mitochondrial complex I deficiency. Am. J. Hum. Genet. 68, 1344–1352. https://doi.org/10.1086/320603

Bénit, P., Slama, A., Cartault, F., Giurgea, I., Chretien, D., Lebon, S., Marsac, C., Munnich, A., Rötig, A., Rustin, P., 2004. Mutant NDUFS3 subunit of mitochondrial complex I causes Leigh syndrome. J. Med. Genet. 41, 14–17.

Berger, I., Hershkovitz, E., Shaag, A., Edvardson, S., Saada, A., Elpeleg, O., 2008. Mitochondrial complex I deficiency caused by a deleterious NDUFA11 mutation. Ann. Neurol. 63, 405–408. https://doi.org/10.1002/ana.21332

Bianciardi, L., Imperatore, V., Fernandez-Vizarra, E., Lopomo, A., Falabella, M., Furini, S., Galluzzi, P., Grosso, S., Zeviani, M., Renieri, A., Mari, F., Frullanti, E., 2016. Exome sequencing coupled with mRNA analysis identifies NDUFAF6 as a Leigh gene. Mol. Genet. Metab. 119, 214–222. https://doi.org/10.1016/j.ymgme.2016.09.001

Calvo, S.E., Compton, A.G., Hershman, S.G., Lim, S.C., Lieber, D.S., Tucker, E.J., Laskowski, A., Garone, C., Liu, S., Jaffe, D.B., Christodoulou, J., Fletcher, J.M., Bruno, D.L., Goldblatt, J., Dimauro, S., Thorburn, D.R., Mootha, V.K., 2012. Molecular diagnosis of infantile mitochondrial disease with targeted next-generation sequencing. Sci. Transl. Med. 4, 118ra10. https://doi.org/10.1126/scitranslmed.3003310

Calvo, S.E., Tucker, E.J., Compton, A.G., Kirby, D.M., Crawford, G., Burtt, N.P., Rivas, M., Guiducci, C., Bruno, D.L., Goldberger, O.A., Redman, M.C., Wiltshire, E., Wilson, C.J., Altshuler, D., Gabriel, S.B., Daly, M.J., Thorburn, D.R., Mootha, V.K., 2010. High-throughput, pooled sequencing identifies mutations in NUBPL and FOXRED1 in human complex I deficiency. Nat. Genet. 42, 851–858. https://doi.org/10.1038/ng.659

Dunning, C.J.R., McKenzie, M., Sugiana, C., Lazarou, M., Silke, J., Connelly, A., Fletcher, J.M., Kirby, D.M., Thorburn, D.R., Ryan, M.T., 2007. Human CIA30 is involved in the early assembly of mitochondrial complex I and mutations in its gene cause disease. EMBO J. 26, 3227–3237. https://doi.org/10.1038/sj.emboj.7601748

Fernandez-Moreira, D., Ugalde, C., Smeets, R., Rodenburg, R.J.T., Lopez-Laso, E., Ruiz-Falco, M.L., Briones, P., Martin, M.A., Smeitink, J.A.M., Arenas, J., 2007. X-linked NDUFA1 gene mutations associated with mitochondrial encephalomyopathy. Ann. Neurol. 61, 73–83. https://doi.org/10.1002/ana.21036

Friederich, M.W., Erdogan, A.J., Coughlin, C.R., Elos, M.T., Jiang, H., O’Rourke, C.P., Lovell, M.A., Wartchow, E., Gowan, K., Chatfield, K.C., Chick, W.S., Spector, E.B., Van Hove, J.L.K., Riemer, J., 2017. Mutations in the accessory subunit NDUFB10 result in isolated complex I deficiency and illustrate the critical role of intermembrane space import for complex I holoenzyme assembly. Hum. Mol. Genet. 26, 702–716. https://doi.org/10.1093/hmg/ddw431

Gerards, M., Sluiter, W., van den Bosch, B.J.C., de Wit, L.E.A., Calis, C.M.H., Frentzen, M., Akbari, H., Schoonderwoerd, K., Scholte, H.R., Jongbloed, R.J., Hendrickx, A.T.M., de Coo, I.F.M., Smeets, H.J.M., 2010. Defective complex I assembly due to C20orf7 mutations as a new cause of Leigh syndrome. J. Med. Genet. 47, 507–512. https://doi.org/10.1136/jmg.2009.067553

Haack, T.B., Danhauser, K., Haberberger, B., Hoser, J., Strecker, V., Boehm, D., Uziel, G., Lamantea, E., Invernizzi, F., Poulton, J., Rolinski, B., Iuso, A., Biskup, S., Schmidt, T., Mewes, H.-W., Wittig, I., Meitinger, T., Zeviani, M., Prokisch, H., 2010. Exome sequencing identifies ACAD9 mutations as a cause of complex I deficiency. Nat. Genet. 42, 1131–1134. https://doi.org/10.1038/ng.706

Haack, T.B., Madignier, F., Herzer, M., Lamantea, E., Danhauser, K., Invernizzi, F., Koch, J., Freitag, M., Drost, R., Hillier, I., Haberberger, B., Mayr, J.A., Ahting, U., Tiranti, V., Rötig, A., Iuso, A., Horvath, R., Tesarova, M., Baric, I., Uziel, G., Rolinski, B., Sperl, W., Meitinger, T., Zeviani, M., Freisinger, P., Prokisch, H., 2012. Mutation screening of 75 candidate genes in 152 complex I deficiency cases identifies pathogenic variants in 16 genes including NDUFB9. J. Med. Genet. 49, 83–89. https://doi.org/10.1136/jmedgenet-2011-100577

Hoefs, S.J.G., Dieteren, C.E.J., Distelmaier, F., Janssen, R.J.R.J., Epplen, A., Swarts, H.G.P., Forkink, M., Rodenburg, R.J., Nijtmans, L.G., Willems, P.H., Smeitink, J.A.M., van den Heuvel, L.P., 2008. NDUFA2 complex I mutation leads to Leigh disease. Am. J. Hum. Genet. 82, 1306–1315. https://doi.org/10.1016/j.ajhg.2008.05.007

Hoefs, S.J.G., van Spronsen, F.J., Lenssen, E.W.H., Nijtmans, L.G., Rodenburg, R.J., Smeitink, J.A.M., van den Heuvel, L.P., 2011. NDUFA10 mutations cause complex I deficiency in a patient with Leigh disease. Eur. J. Hum. Genet. 19, 270–274. https://doi.org/10.1038/ejhg.2010.204

Kirby, D.M., Salemi, R., Sugiana, C., Ohtake, A., Parry, L., Bell, K.M., Kirk, E.P., Boneh, A., Taylor, R.W., Dahl, H.-H.M., Ryan, M.T., Thorburn, D.R., 2004. NDUFS6 mutations are a novel cause of lethal neonatal mitochondrial complex I deficiency. J. Clin. Invest. 114, 837–845. https://doi.org/10.1172/JCI20683

Kohda, M., Tokuzawa, Y., Kishita, Y., Nyuzuki, H., Moriyama, Y., Mizuno, Y., Hirata, T., Yatsuka, Y., Yamashita-Sugahara, Y., Nakachi, Y., Kato, H., Okuda, A., Tamaru, S., Borna, N.N., Banshoya, K., Aigaki, T., Sato-Miyata, Y., Ohnuma, K., Suzuki, T., Nagao, A., Maehata, H., Matsuda, F., Higasa, K., Nagasaki, M., Yasuda, J., Yamamoto, M., Fushimi, T., Shimura, M., Kaiho-Ichimoto, K., Harashima, H., Yamazaki, T., Mori, M., Murayama, K., Ohtake, A., Okazaki, Y., 2016. A Comprehensive Genomic Analysis Reveals the Genetic Landscape of Mitochondrial Respiratory Chain Complex Deficiencies. PLoS Genet. 12, e1005679. https://doi.org/10.1371/journal.pgen.1005679

Kremer, L.S., Bader, D.M., Mertes, C., Kopajtich, R., Pichler, G., Iuso, A., Haack, T.B., Graf, E., Schwarzmayr, T., Terrile, C., Koňaříková, E., Repp, B., Kastenmüller, G., Adamski, J., Lichtner, P., Leonhardt, C., Funalot, B., Donati, A., Tiranti, V., Lombes, A., Jardel, C., Gläser, D., Taylor, R.W., Ghezzi, D., Mayr, J.A., Rötig, A., Freisinger, P., Distelmaier, F., Strom, T.M., Meitinger, T., Gagneur, J., Prokisch, H., 2017. Genetic diagnosis of Mendelian disorders via RNA sequencing. Nat. Commun. 8, 15824. https://doi.org/10.1038/ncomms15824

Loeffen, J., Elpeleg, O., Smeitink, J., Smeets, R., Stöckler-Ipsiroglu, S., Mandel, H., Sengers, R., Trijbels, F., van den Heuvel, L., 2001. Mutations in the complex I NDUFS2 gene of patients with cardiomyopathy and encephalomyopathy. Ann. Neurol. 49, 195–201.

Loeffen, J., Smeitink, J., Triepels, R., Smeets, R., Schuelke, M., Sengers, R., Trijbels, F., Hamel, B., Mullaart, R., van den Heuvel, L., 1998. The first nuclear-encoded complex I mutation in a patient with Leigh syndrome. Am. J. Hum. Genet. 63, 1598–1608. https://doi.org/10.1086/302154

Ogilvie, I., Kennaway, N.G., Shoubridge, E.A., 2005. A molecular chaperone for mitochondrial complex I assembly is mutated in a progressive encephalopathy. J. Clin. Invest. 115, 2784–2792. https://doi.org/10.1172/JCI26020

Ostergaard, E., Rodenburg, R.J., van den Brand, M., Thomsen, L.L., Duno, M., Batbayli, M., Wibrand, F., Nijtmans, L., 2011. Respiratory chain complex I deficiency due to NDUFA12 mutations as a new cause of Leigh syndrome. J. Med. Genet. 48, 737–740. https://doi.org/10.1136/jmg.2011.088856

Piekutowska-Abramczuk, D., Assouline, Z., Mataković, L., Feichtinger, R.G., Koňařiková, E., Jurkiewicz, E., Stawiński, P., Gusic, M., Koller, A., Pollak, A., Gasperowicz, P., Trubicka, J., Ciara, E., Iwanicka-Pronicka, K., Rokicki, D., Hanein, S., Wortmann, S.B., Sperl, W., Rötig, A., Prokisch, H., Pronicka, E., Płoski, R., Barcia, G., Mayr, J.A., 2018. NDUFB8 Mutations Cause Mitochondrial Complex I Deficiency in Individuals with Leigh-like Encephalomyopathy. Am. J. Hum. Genet. 102, 460–467. https://doi.org/10.1016/j.ajhg.2018.01.008

Potluri, P., Davila, A., Ruiz-Pesini, E., Mishmar, D., O’Hearn, S., Hancock, S., Simon, M., Scheffler, I.E., Wallace, D.C., Procaccio, V., 2009. A novel NDUFA1 mutation leads to a progressive mitochondrial complex I-specific neurodegenerative disease. Mol. Genet. Metab. 96, 189–195. https://doi.org/10.1016/j.ymgme.2008.12.004

Saada, A., Edvardson, S., Rapoport, M., Shaag, A., Amry, K., Miller, C., Lorberboum-Galski, H., Elpeleg, O., 2008. C6ORF66 is an assembly factor of mitochondrial complex I. Am. J. Hum. Genet. 82, 32–38. https://doi.org/10.1016/j.ajhg.2007.08.003

Saada, A., Vogel, R.O., Hoefs, S.J., van den Brand, M.A., Wessels, H.J., Willems, P.H., Venselaar, H., Shaag, A., Barghuti, F., Reish, O., Shohat, M., Huynen, M.A., Smeitink, J.A.M., van den Heuvel, L.P., Nijtmans, L.G., 2009. Mutations in NDUFAF3 (C3ORF60), encoding an NDUFAF4 (C6ORF66)-interacting complex I assembly protein, cause fatal neonatal mitochondrial disease. Am. J. Hum. Genet. 84, 718–727. https://doi.org/10.1016/j.ajhg.2009.04.020

Sánchez-Caballero, L., Ruzzenente, B., Bianchi, L., Assouline, Z., Barcia, G., Metodiev, M.D., Rio, M., Funalot, B., van den Brand, M.A.M., Guerrero-Castillo, S., Molenaar, J.P., Koolen, D., Brandt, U., Rodenburg, R.J., Nijtmans, L.G., Rötig, A., 2016. Mutations in Complex I Assembly Factor TMEM126B Result in Muscle Weakness and Isolated Complex I Deficiency. Am. J. Hum. Genet. 99, 208–216. https://doi.org/10.1016/j.ajhg.2016.05.022

Smeitink, J., van den Heuvel, L., 1999. Human mitochondrial complex I in health and disease. Am. J. Hum. Genet. 64, 1505–1510. https://doi.org/10.1086/302432

Spiegel, R., Shaag, A., Mandel, H., Reich, D., Penyakov, M., Hujeirat, Y., Saada, A., Elpeleg, O., Shalev, S.A., 2009. Mutated NDUFS6 is the cause of fatal neonatal lactic acidemia in Caucasus Jews. Eur. J. Hum. Genet. EJHG 17, 1200–1203. https://doi.org/10.1038/ejhg.2009.24

van den Bosch, B.J.C., Gerards, M., Sluiter, W., Stegmann, A.P.A., Jongen, E.L.C., Hellebrekers, D.M.E.I., Oegema, R., Lambrichs, E.H., Prokisch, H., Danhauser, K., Schoonderwoerd, K., de Coo, I.F.M., Smeets, H.J.M., 2012. Defective NDUFA9 as a novel cause of neonatally fatal complex I disease. J. Med. Genet. 49, 10–15. https://doi.org/10.1136/jmedgenet-2011-100466

van den Heuvel, L., Ruitenbeek, W., Smeets, R., Gelman-Kohan, Z., Elpeleg, O., Loeffen, J., Trijbels, F., Mariman, E., de Bruijn, D., Smeitink, J., 1998. Demonstration of a new pathogenic mutation in human complex I deficiency: a 5-bp duplication in the nuclear gene encoding the 18-kD (AQDQ) subunit. Am. J. Hum. Genet. 62, 262–268. https://doi.org/10.1086/301716

Table S2: Composition of individual MEGS incubations.

| incubation | Substrates |
| --- | --- |
| 1 | [1-^14^C]pyruvate + malate + ADP |
| 2 | [1-^14^C]pyruvate + carnitine + ADP |
| 3 | [1-^14^C]pyruvate + malate **without**ADP |
| 4 | [1-^14^C]pyruvate + malate **without**ADP + CCCP |
| 4a | [1-^14^C]pyruvate**without**carnitine + ADP |
| 5 | [1-^14^C]pyruvate + malate + ADP + atractyloside |
| 6 | [U-^14^C]malate + pyruvate + malonate + ADP |
| 7 | [U-^14^C]malate + acetylcarnitine + malonate  + ADP |
| 8 | [U-^14^C]malate + acetylcarnitine + arsenite + ADP |
| 9 | [1,4-^14^C]succinate + acetylcarnitine + ADP |
